# Selenium and Coenzyme Q_10_ Supplementation and Sex Differences in Cardiovascular Mortality Results from a Prospective Randomized Double-Blind Placebo-Controlled Trial in Elderly People Low in Selenium

**DOI:** 10.3390/antiox14060685

**Published:** 2025-06-05

**Authors:** Urban Alehagen, Jan Olav Aaseth, Lutz Schomburg, Trine B. Opstad, Anders Larsson, Jan Alexander

**Affiliations:** 1 Division of Cardiovascular Medicine, Department of Medical and Health Sciences, Linköping University, SE-581 85 Linköping, Sweden; 2 Research Department, Innlandet Hospital Trust, N-2381 Brumunddal, Norway; jaol-aas@online.no; 3 Institute for Experimental Endocrinology, Charité-Universitätsmedizin Berlin, D-10115 Berlin, Germany; lutz.schomburg@charite.de; 4 Oslo Center for Clinical Heart Research Laboratory, Department of Cardiology, Oslo University Hospital Ullevål, N-0450 Oslo, Norway; t.b.opstad@medisin.uio.no; 5 Faculty of Medicine, University of Oslo, N-0372 Oslo, Norway; 6 Department of Medical Sciences, Uppsala University, SE-751 85 Uppsala, Sweden; anders.larsson@medsci.uu.se; 7 Norwegian Institute of Public Health, N-0403 Oslo, Norway; jan.alexander@fhi.no

**Keywords:** selenium, coenzyme Q_10_, elderly, sex differences, cardiovascular mortality

## Abstract

**Background:** Low selenium intake and age-related decline of coenzyme Q_10_ (CoQ_10_) have been associated with an increased risk of cardiovascular disease (CVD) and oxidative stress. In a randomised placebo-controlled trial (RTC) in elderly people with low selenium levels, the supplementation with selenium and CoQ_10_ reduced CVD and mortality. However, whether the supplementation elicited sex-specific benefits remained to be explored. **Methods:** Elderly Swedish persons (*n* = 443; balanced sex ratio) receiving selenium yeast (200 µg/day) and CoQ_10_ (200 mg/day) combined or a placebo for four years were followed for additional six years. The response to supplementation, cardiovascular (CV) mortality, and risk factors were determined at four and ten years. Kaplan–Meier analyses, ANCOVA, repeated measurements of variance, and Cox proportional hazard regression analyses were performed. **Results:** The measured 10-year CV mortality rate was lower in females, and supplementation reduced this risk to a greater extent compared to in males. The improved survival rate apparently kicked in later in females than in males. At baseline, males had a higher smoking rate, increased inflammation and oxidative stress, and a higher prevalence of more advanced ischaemic heart disease (IHD) and signs of heart failure. When stratified by sex, in individuals with IHD, the intervention improved CV survival in both sexes, whereas supplementation had a more pronounced effect in females without IHD at inclusion. Supplementation diminished inflammation and oxidative stress, impaired the increase of NT-proBNP, and improved renal function in both sexes. **Conclusions**: The supplementation improved CV survival, especially in women. The higher prevalence of structural CVD and smoking in males may have contributed to the observed greater supplementation benefits in females. The preventive impact of selenium and CoQ_10_ supplementation in elderly males and females may be particularly strong and meaningful in the early stages of CVD development.

## 1. Introduction

We have previously reported that dietary supplementation with the antioxidants selenium and coenzyme Q_10_ in elderly subjects low in selenium in a randomised placebo-controlled trial lasting for four years substantially decreased CV mortality after five, ten, and twelve years [1,2,3]. Importantly, in those supplemented, we observed a significantly lower level of inflammation and oxidative stress, which are among several major factors in the initiation of atherosclerosis [4,5]. However, it was not known whether the supplementation effects of selenium and coenzyme Q_10_ differ between the sexes.

From the literature, numerous reports have shown differences in the development and character of CVD between the two sexes [6,7,8]. Males tend to suffer from vascular changes early in life, whereas the female group exhibits such changes later, especially after menopause. Vascular stiffness and atherosclerosis are examples of clearly recognisable changes where differences can be seen between the sexes [8]. Even though a meta-analysis reported equal relations between systolic blood pressure and risk for stroke or ischaemic heart disease (IHD), based on 124 prospective population studies including 1.2 million patients [9], several studies have reported a preponderance of males with IHD in comparison with females, such as the IHD analysis from 204 countries between 1990 and 2019 by Safiri et al. [10]. Regarding hypertension, it is well known that the incidence of hypertension increases with age in a sex-specific manner. In middle age, hypertension is more prevalent in males, but after menopause, its prevalence increases substantially in females [11].

Selenium is an essential trace element that occurs as selenocysteine in selenoproteins, playing important roles in i.a. inflammation, protection against oxidative stress, redox regulation, thyroid metabolism, and protein folding.

In many areas of the world, e.g., Europe, the selenium content of the soil is low, with low dietary intake resulting in insufficiency [12,13], contrary to the U.S. [14,15,16,17,18,19,20].

CoQ_10_ is a powerful lipid-soluble antioxidant [21], and it has been shown that coenzyme Q_10_ reduces the inflammatory response [22]. However, the endogenous production of CoQ_10_ decreases after the age of 20, and the content in the heart is reduced to about half at the age of 80 [23]. Selenium and CoQ_10_ work in concert as a selenoenzyme, thioredoxin reductase reduces ubiquinone to ubiquinol, the active form of the coenzyme [24].

It is known from the literature that women and men differ significantly in terms of cardiovascular disease development, prevalence, and mortality [25], and it was of interest to apply this perspective to this study population, in which we found that supplementation with selenium and CoQ_10_ significantly reduced CVD, and mortality during follow-up [1,2,3]. We therefore hypothesized that supplementation might have been differentially effective in men and women, with possible differences depending on the different stages of CVD development.

**Aim:** The aim of this sub-analysis was to explore whether elderly men and women low in selenium responded differently to supplementation with selenium and coenzyme Q_10_ and, if so, to explore possible underlying reasons that may contribute to such a difference.

## 2. Methods

### 2.1. Study Population

In 1998, individuals aged 69 to 88 years living in a rural municipality in south-eastern Sweden (*n* = 1320) were invited to participate in an epidemiological study with a focus on CVD. Of these, 876 consented to take part. In 2003, all surviving participants from the initial group (*n* = 675) were invited to enrol in a randomised controlled intervention study. Among them, 443 community-dwelling individuals displaying a balanced sex ratio chose to participate in the study, which involved a four-year supplementation with selenium and CoQ_10_, or a placebo, with blood samples collected every six months.

The participants received daily dietary supplements of 200 mg of coenzyme Q_10_ in capsule form (Bio-Quinon 100 mg B.I.D, Pharma Nord, Vejle, Denmark) and 200 µg of organic selenium yeast tablets (SelenoPrecise 100 µg B.I.D, Pharma Nord, Vejle, Denmark) (*n* = 221) or a similar placebo (*n* = 222) over 48 months. This supplementation was in addition to any regular medications they were taking. To measure compliance, all unused study medications (both active and placebo) were returned and counted, with no significant difference in unused medications between the two groups. The participants were assessed by one of three experienced cardiologists, who recorded clinical histories and conducted examinations at both the beginning and the end of the study. Blood pressure was measured, and evaluations included the New York Heart Association (NYHA) functional class, electrocardiograms (ECGs), and Doppler echocardiography. Echocardiographic tests were performed with the participants in the left lateral position, and ejection fraction (EF) readings were categorised into four classes based on limits of 30%, 40%, and 50%. Normal systolic function was defined as EF ≥ 50%, while severely impaired systolic function was indicated by EF < 30%. Only systolic function was assessed. The inclusion phase ran from January 2003 to February 2010. A participant was classified as having IHD if they (1) had survived a previous myocardial infarction, (2) had symptomatic angina pectoris, or (3) had a pathological ECG indicating ischaemic heart disease.

### 2.2. Ethical Approval

The research received approval from the Regional Ethics Committee (Forskningsetikkommmitten, Hälsouniversitetet, SE-581 85 Linköping, Sweden; No. D03-176) and adhered to the ethical principles outlined in the 1975 Declaration of Helsinki. The Medical Product Agency did not review the study protocol as it did not involve testing a medication for a specific disease but rather examined commercially available food supplements. Registration for this study took place on Clinicaltrials.gov under the identifier NCT01443780, although this was carried out retrospectively since registration was not compulsory when the study started. All participants provided written and informed consent before their involvement.

### 2.3. Blood Sampling

Participants’ blood samples were obtained upon enrolment and after 42 and 48 months while they were in a supine position. Whole blood samples were gathered in Vacutainer tubes and serum preparation vials underwent centrifugation at 3000 g, +4 °C. For EDTA plasma and citrate plasma, pre-chilled EDTA vials and sodium citrate vials (0.11 mol/L) were used. The vials were centrifuged at 3000 g, +4 °C. Whole blood, serum, and plasma were frozen at −70 °C until analysis, ensuring that samples were only thawed twice at most.

### 2.4. Determination of Selenium

The serum selenium assessments were conducted utilising ICP-MS methodology on an Agilent 700 platform at Kompetenzzentrum für komplementärmedizinische Diagnostik, Zweigniederlassung der synlab MVZ Leinfelden GmbH (Leinfelden-Echterdingen, Germany), as described [26]. Accuracy verification was performed by analysing two external reference materials with certified values of 63 μg/L and 103 μg/L through a control programme run by the Society for Advancement of Quality Assurance in Medical Laboratories, INSTAND e.V., Düsseldorf, Germany, displaying results within 90–110% of certified concentrations. Method precision assessment, carried out by repetitive analyses of identical sera, displayed an average coefficient of variation of 5.7%.

### 2.5. Determination of Biomarkers

The chosen biomarkers were previously determined in plasma. Those performing the measurements were blinded as to the purpose of the study and had no knowledge of the clinical data.

### 2.6. Determination of P-Selectin and Osteopontin

Soluble P-selectin (sP-selectin, sCD62P) was analysed utilising an ELISA from R&D (Abingdon, UK). The intra-assay coefficient of variation was about 5%, and the inter-assay coefficient of variation was about 9%. Plasma osteopontin was analysed using a commercial ELISA kit (DY1433, R&D Systems, Minneapolis, MN, USA). The assay had a total coefficient of variation of approximately 6%.

### 2.7. Determination of NT-proBNP and Copeptin

ProBNP 1-76 (NT-proBNP) was measured on the Elecsys 2010 platform (Roche Diagnostics, Mannheim, Germany). The total coefficient of variation was 4.8% at 26 pmol/L and 2.1% at 503 pmol/L. Plasma copeptin was measured on the Kryptor Compact platform (BRAHMS GmbH, Hennigsdorf, Germany). The inter-assay coefficients of variation are <15% at 20 pmol/L, <13% for 20–50 pmol/L, and <8% for concentrations >50 pmol/L according to previous validation [27] and information from the manufacturer [27].

### 2.8. Parameter of Renal Function

It is well-known that the serum concentration of creatinine is influenced by the total muscle mass of the individual, which in this population of elderly people could change substantially over the four years of intervention. Renal function is also influenced by gender and age. As cystatin-C is not strongly related to muscle mass, it is considered a better measure of kidney function in the elderly. Cystatin-C was therefore also determined.

To adjust for sex, age, and race, appropriate algorithms have been proposed. Here we applied the most widely used algorithm, the Chronic Kidney Disease Epidemiology Collaboration (CKD-Epi) algorithm, which combines both creatinine and cystatin-C and is also appropriate for those with low glomerular filtration rates (GFRs) [28]. The creatinine factor for African Americans has not been used in the calculations.

### 2.9. Statistical Methods

Descriptive data are expressed as percentages or as means ± standard deviation (SD). A Student’s unpaired two-sided *t*-test was employed to analyse continuous variables, while the chi-square test was used for categorical variable analysis. Repeated measures of variance were utilised to provide more detailed information on individual changes in biomarker concentrations compared to group averages.

To assess the potential association between selenium and CoQ_10_ supplementation and serum levels of various inflammatory and oxidative stress biomarkers, Pearson’s product–moment correlation analysis was conducted. The influence of covariates, apart from the active treatment, on different biomarkers at the 48-month mark was evaluated using analysis of covariance (ANCOVA). Kaplan–Meier and Cox proportional hazards regression analyses were applied to assess CV mortality during the follow-up period.

The delta values of osteopontin were obtained by subtracting the concentration value after 42 months from the value at inclusion.

*p*-values < 0.05 were considered significant, based on a two-sided evaluation. All data were analysed using standard software (Statistica v. 13.2, Dell Inc., Tulsa, OK, USA).

## 3. Results

### 3.1. Baseline Characteristics of the Active Treatment Versus the Placebo Groups

The main study population consisted of 443 participants with a balanced sex ratio. Of those, 221 received active supplementation with selenium and CoQ_10_, whereas 222 participants received the placebo. (For the CONSORT Flow diagram, see the Supplemental Figure S1). The two groups were well balanced with regard to anthropometrics, history, and medication, as illustrated in Table 1.

Except for the use of ACE inhibitors, there were no significant differences between the two groups.

### 3.2. Serum Selenium Concentration in Males and Females

The pre-intervention selenium status of the total study population was about 67.7 ± 15.0 µg/L (Table 1), indicating that a low status, a value above 100 µg/L, is required for full expression of selenoenzymes [29]. Upon comparing the serum selenium concentration in the two groups of males and females, a slightly higher concentration of selenium was observed in the females, as compared with the males (females: 70.0 µg/L, SD: 15.6 vs. males: 65.6 µg/L, SD: 14.9; *p* = 0.03), which is in accordance with the literature [30,31]. We have previously found that the increase in selenium after supplementation (Table 1 and Table 3) was accompanied by a maximised expression of circulating Selenoprotein P and Glutathione peroxidase 3 [32,33].

### 3.3. Mortality and Sex Differences

As the objective of this study was to assess if any difference could be seen between males and females in the effectiveness of supplementation with selenium and CoQ_10_ versus a placebo, mortality was chosen as a hard endpoint and evaluated up to 10 years after inclusion in the main study. The intervention time was four years, and the follow-up included six more years without active intervention in the two groups. During the period between when the intervention was stopped and the follow-up after 10 years, no supplementation was provided. Two years after the intervention was stopped, all participants were interviewed to record if they had bought selenium and CoQ_10_ on their own. The proportion of those who bought supplements on their own were equal between the active intervention and placebo groups (and between males and females).

Evaluating the female group for CV mortality after 10 years, we found 12 deaths out of 106 (11.3%) in the active treatment group, compared with 37 deaths out of 112 in the placebo group (33.0%; Chi2: 14.74; *p* = 0.0001).

In the male group, 34 deaths out of 115 (29.6%) in the active treatment group were found, and 49 deaths out of 110 were found in the placebo group (44.5%; Chi2: 5.42; *p* = 0.02). The active intervention was associated with a reduction in mortality of about 2/3 in women and 1/3 in men.

**Figure 1:**
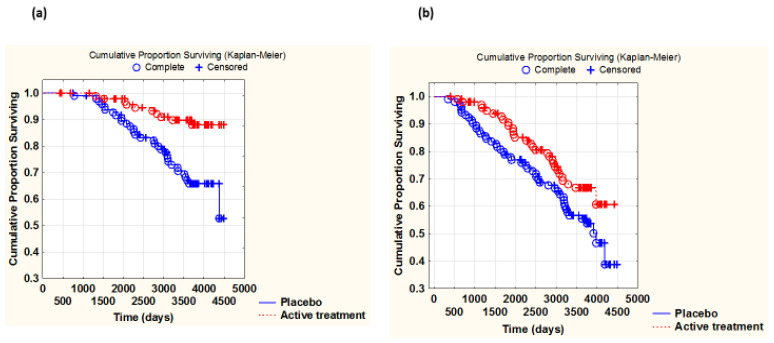
Kaplan–Meier graph illustrating survival from CV mortality in the female study population (**a**) and in the male population (**b**), comparing those with active supplementation versus those on placebo during a follow-up period of 10 years. (**a**) Z = −3.52, *p* = 0.00004. Active treatment: *n* = 106; placebo: *n* = 112. (**b**) −2.03, *p* = 0.042. Active treatment: *n* = 115; placebo: *n* = 110.

Similarly, from the Kaplan–Meier analyses, the active intervention evidently had a greater impact on CV mortality in women than in men (the difference between the placebo and actively treated groups (Z-score) was greater in women as compared with that in men) (females: Z = −3.52; *p* = 0.0004 vs. males: Z = −2.03; *p* = 0.042) (Figure 1a,b).

Evaluating the risk of suffering CV mortality in relation to some well-known risk factors, active treatment produced a significantly reduced risk in both sexes, although it was apparently greater in the female group (hazard ratio: females: 0.29; males: 0.61) (Table 2) and similar, as indicated above: by about 2/3 in the female group and 1/3 in the male group. As can be seen in Table 2, IHD was the strongest risk factor for females, in addition to high NT-proBNP levels, whereas high NT-proBNP and smoking were the highest risk factors for men, in addition to the presence of diabetes in both sexes. The number of smokers was balanced between the placebo and actively treated groups when evaluating CV mortality following stratification into males and females and IHD and non-IHD.

### 3.4. Baseline Characteristics of the Two Sexes

To shed some light on the potentially underlying factors of relevance to the extensive differences in CV mortality, we stratified the characteristics at baseline according to sex (Table 3).

**Table 3:** Baseline characteristics of the study population divided into females and males receiving active treatment or placebo during an intervention time of four years.

	Females *n* = 218	Males *n* = 225	*p*-Value
**Age years, mean (SD)**	77.0 (3.0)	77.3 (3.3)	0.36
**History**			
**Smoking, *n* (%)**	9 (4.1)	32 (14.2)	0.0002
**Hypertension, *n* (%)**	165 (75.7)	162 (72.0)	0.38
**IHD, *n* (%)**	38 (17.4)	62 (27.6)	0.01
**Diabetes, *n* (%)**	46 (21.1)	50 (22.2)	0.77
**Obstr pulm disease, *n* (%)**	31(14.2)	27 (12.0)	0.49
**NYHA class I, *n* (%)**	116 (53.2)	110 (48.9)	0.36
**NYHA class II, *n* (%)**	56 (25.7)	68 (30.2)	0.29
**NYHA class III, *n* (%)**	42 (19.3)	46 (20.4)	0.68
**Unclassified, *n* (%)**	3	1	-
**BMI, mean (SD)**	27.6 (5.3)	26.7 (3.2)	0.04
**Medications**			
**Beta blockers, *n* (%)**	72 (33.0)	82 (33.7)	0.41
**ACEI/ARB, *n* (%)**	53 (24.3)	59 (26.2)	0.66
**Digitalis, *n* (%)**	13 (6.0)	9 (4.0)	0.34
**Diuretics, *n* (%)**	91 (41.7)	67 (29.8)	0.009
**Statins, *n* (%)**	32(14.7)	46 (20.4)	0.007
**Nitrates, *n* (%)**	28 (12.8)	50 (22.2)	0.01
**Examinations**			
**CRP mg/L mean, (SD)**	3.9 (5.8)	6.0 (15.8)	0.07
**CKD-Epi mL/min/1.73 m^2^**	61.5 (18.4)	61.7 (15.3)	0.94
**HDL mmol/L mean, (SD)**	1.72 (0.43)	1.35 (0.31)	<0.0001
**LDL mmol/L mean, (SD)**	4.04 (1.06)	3.73 (0.83)	0.005
**Triglycerides mmol/L mean, (SD)**	1.35 (0.56)	1.35 (0.65)	0.91
**EF < 40%, *n* (%)**	9 (4.1)	24 (10.7)	0.009
**NT-proBNP ng/L mean (SD)**	419 (649)	658 (1662)	0.048
**s-Se pre-intervention µg/L, mean (SD)**	70.0 (15.6)	65.6 (14.9)	0.03
**s-Se post-intervention µg/L, mean (SD)**	224.8 (68.6)	210.8 (45.0)	0.38

Note: ACEI: ACE inhibitors; ARB: angiotensin receptor blockers; BMI: body mass index; CKD-Epi: the Chronic Kidney Disease Epidemiology Collaboration (CKD-Epi) algorithm; EF: ejection fraction; IHD: ischaemic heart disease; NT-proBNP: N-terminal fragment of B-type natriuretic peptide; NYHA: New York Heart Association functional class; SD: standard deviation; s-Se: serum selenium. Values are means ± SDs or frequency (percent). Student’s unpaired two-sided *t*-test was used for continuous variables, and the chi-square test was used for analysis of one discrete variable.

From Table 3, it was evident that the male group demonstrated important differences compared with the females. The male group consisted of significantly more smokers compared to the female group. Also, significantly more males had a history of IHD than did the female group, and, as a result, more males were being treated with statins and nitrates. More males had impaired cardiac function according to the echocardiography and also signs of increased cardiac wall tension, as seen from the concentration of NT-proBNP.

Based on the differences between males and females above, we hypothesised that the supplementation might have less impact on CV mortality in participants with more advanced CVD at inclusion, such as IHD. We therefore evaluated CV mortality in males and females with diagnosed IHD. From the *t*-tests, we found a clear indication of less CV mortality among those actively treated in both sexes, (females: active: 7 CV deaths out of 20 (35.0%); placebo: 13 CV deaths out of 18 (72.2%); *p* = 0.02; males: active: 9 CV deaths out of 27 (33.3%); placebo: 20 CV deaths out of 35 (57.1%); *p* = 0.06). A reduction of deaths of about 50% was found in both sexes.

By evaluating the impact of active treatment on CV mortality in those without diagnosed IHD, the results indicated a stronger reducing effect of active treatment among females than among males: (females: active treatment: 4 CV deaths out of 86 (4.7%); placebo: 27 CV deaths out of 94 (28.7%); *p* < 0.0001; males: active treatment: 21 CV deaths out of 88 (23.7%); placebo: 30 CV deaths out of 75 (40.0%); *p* = 0.03).

Due to the limited sample size of the subgroups and to further explore the pattern of CV survival in the actively treated and placebo groups, we combined the two sexes and performed a Kaplan–Meier analysis of CV mortality of participants with and without IHD at inclusion. As mentioned above, the numbers of smokers were balanced when evaluating the active treatment versus the placebo groups in the IHD and in the non-IHD groups.

**Figure 2:**
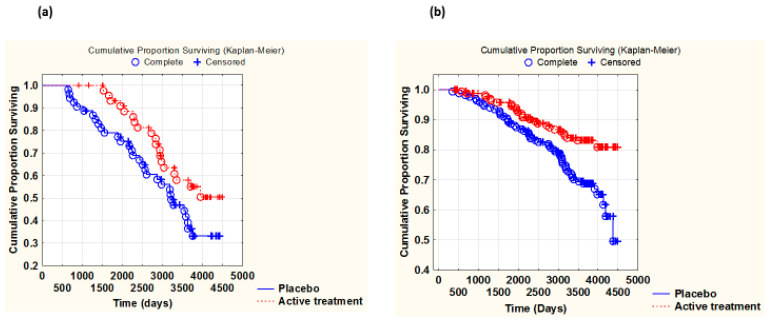
Kaplan–Meier graph illustrating survival from CV mortality in the group with IHD at inclusion (**a**) and in the non-IHD group at inclusion (**b**). Males and females combined, comparing those with active supplementation versus those on placebo, during a follow-up period of 10 years. (**a**) Z = −2.20, *p* = 0.03. Active treatment: *n*= 47; placebo: *n* = 53. (**b**) Z =−2.82; *p* = 0.04. Active treatment: *n* = 174; placebo: *n* = 169.

Among those with IHD at inclusion, we found significantly less CV mortality among those who received active supplementation with selenium and CoQ_10,_ compared with the placebo (Z = −2.20; *p* = 0.03). Also, among those without IHD, we found that supplementation reduced CV mortality (z = −2.82; *p* = 0.04) (Figure 2a,b).

However, the Kaplan–Meier analysis showed striking differences. While in those with IHD, active treatment seemed to increase survival during the whole follow-up period, among those without IHD, increased survival appeared to kick in past the active intervention period, at about 1500 to 1800 days or 48 to 60 months.

### 3.5. Inflammation and the Two Sexes

#### 3.5.1. C-Reactive Protein (CRP)

CRP is one of the most well-known biomarkers of inflammation, and it was evaluated in the two sexes. At inclusion, females had a lower CRP value than males, although not significantly (females: 3.9 mg/L, males 6.0 mg/L, *p* = 0.07). No significant difference in CRP levels at baseline could be noted between the placebo versus the active treatment groups in either the female or the male groups (females: active treatment group: 4.43 mg/L; placebo: 3.93 mg/L; *p* = 0.49; males: active treatment group: 6.68 mg/L; placebo: 10.76 mg/L; *p* = 0.12).

After 48 months, no significant difference was seen in the female group (females: active treatment group: 3.86 mg/L; placebo: 4.18 mg/L; *p* = 0.74), while among the males, the active group had a significantly lower CRP concentrations than the placebo group (active treatment group: 6.04 mg/L; placebo: 10.76 mg/L; *p* = 0.014).

**Figure 3:**
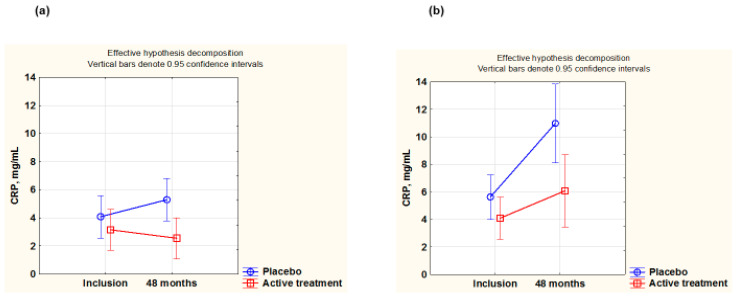
CRP concentration evaluated in the active treatment group versus placebo group at inclusion and after 48 months in the female group (**a**) and in the male group (**b**). Evaluation performed by use of repeated measures of variance methodology. Note: Vertical bars denote 0.95 confidence interval. Blue curve: placebo; red curve: active treatment. Note: (**a**) Current effect: F(1,113) = 5.39; *p* = 0.022. Active treatment: *n* = 60; placebo: *n* = 55. (**b**) Current effect: F(1,94) = 4.05; *p* = 0.047. Active treatment: *n* = 55; placebo: *n* = 45.

Next, repeated measurement of variance (using the population surviving for 48 months) was applied to better follow the change in each individual. Significant differences were found in both the male and the female groups, (females: F(1,113)= 5.39; *p* = 0.022; males: F(1,94) = 4.05; *p* = 0.047). A smaller increase in CRP levels could be seen in the active treatment group (Figure 3a,b).

#### 3.5.2. P-Selectin

As P-selectin is also a biomarker for inflammation, the concentration of the biomarker was evaluated in the two groups. At inclusion, the females had a lower P-selectin concentration than the males (females: 56.3 ng/mL; males: 62.3 ng/mL; *p* = 0.012). However, when comparing the active treatment and the placebo groups in the female and male groups at inclusion, no significant differences in P-selectin levels were present (females: active treatment group: 57.0 ng/mL; placebo: 55.7 ng/mL; *p* = 0.70; males: active treatment group: 59 ng/mL; placebo: 65.6 ng/mL; *p* = 0.07).

**Figure 4:**
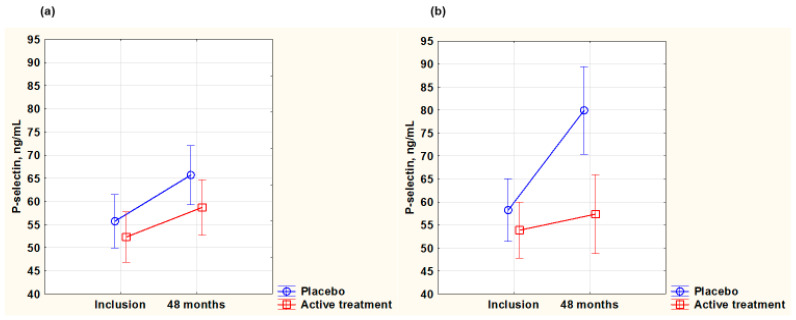
P-selectin concentration evaluated in the active treatment group versus placebo group at inclusion and after 48 months in the female group (**a**) and in the male group (**b**). Note: (**a**) Current effect: F(1,115) = 0.53; *p* = 0.47. Active treatment: *n* = 62; placebo: *n* = 56. (**b**) Current effect: F(1,97) = 10.59; *p* = 0.002. Active treatment: *n* = 56; placebo: *n* = 44. Evaluation performed by use of repeated measures of variance methodology. Vertical bars denote 0.95 confidence interval. Blue curve: placebo; red curve: active treatment.

After 48 months, in the female group, no difference was noted between the active treatment and placebo groups (active: 59.2 ng/mL; placebo: 67.6 ng/mL; *p* = 0.09). Upon applying repeated measures of variance (evaluating only participants surviving for four years), there was no change in the groups during the intervention period, and no significant difference between the active and the placebo groups was detected (*p* = 0.47) (Figure 4a).

However, in the male group after 48 months, the *p*-selectin concentration was significantly higher in the placebo group than in the active treatment group (active: 59.3 ng/mL; placebo: 77.9 ng/mL; *p* = 0.01). Upon applying repeated measures of variance, the value of the placebo group seemed to increase substantially during the intervention period and showed a significantly higher value than the active group, which remained stable over time (*p* = 0.002) (Figure 4b).

#### 3.5.3. Osteopontin

Osteopontin is another biomarker for inflammation that was evaluated in the two sexes. At inclusion, there was no difference in the osteopontin concentration between females and males (females: 67.4 ng/mL; males: 63.6 ng/mL; *p* = 0.47).

In the female group, there was no significant difference between the active treatment group and the placebo group at inclusion (active: 69.53 ng/mL; placebo: 65.33 ng/mL; *p* = 0.66). In the male group, equally, there was no significant difference between the active treatment and the placebo groups at inclusion (active: 62.23 ng/mL; placebo: 65.48 ng/mL; *p* = 0.41). However, after 42 months, a highly significant difference was observed in the female group: a lower level in the active group (active: 56.02 ng/mL; placebo: 67.58 ng/mL; *p* = 0.002). In the male group after 42 months, a greater increase in osteopontin concentration was noted in the placebo group, compared with the active treatment group (active: 64.18 ng/mL; placebo: 77.10 ng/mL; *p* = 0.01).

To examine this further, both between the two sexes and between the placebo and active groups within the same sex, the delta values (osteopontin_incl_.−osteopontin_42m._) for each participant surviving 42 months were analysed. In the female group, a large positive delta value was seen in the active treatment group, whereas there was a weak negative value in the placebo group (active: 13.51; placebo: −2.25; T-value: −1.63; *p* = 0.11). Active treatment apparently reduced inflammation in females. In the male group, different results were seen: in the active group, there was a weak negative delta value, while in the placebo group, there was a strong negative delta (active: −1.95; placebo: −11.62; T-value: −2.18; *p*= 0.03). This indicates that in males receiving the placebo, inflammation increased substantially during the intervention period, while supplementation effectively prevented an increase.

### 3.6. Oxidative Stress and the Two Sexes

#### 3.6.1. Copeptin

As copeptin is also a biomarker for oxidative stress, among other functions, we evaluated this biomarker in the two sexes. At inclusion, the copeptin value was significantly higher in males than in females (males 16.5 pmol/L, females 8.7 pmol/L, *p* < 0.0001), indicating a higher level of oxidative stress in males.

In the female group, no significant differences in the concentration of copeptin were noted at inclusion (active: 8.2 pmol/L; placebo: 9.1 pmol/L; *p* = 0.43). After 48 months, there was no difference between the two groups (active: 12.6 pmol/L; placebo: 9.5 pmol/L; *p* = 0.18). In the male group, no difference in the concentration of copeptin was found at inclusion (active: 16.9 pmol/L; placebo: 16.1 pmol/L; *p* = 0.76), and both groups had higher values than the corresponding ones for females. After 48 months, a slightly higher value in the placebo group, although not statistically significant, could be noted (active: 17.8 pmol/L; placebo: 20.0 pmol/L; *p* = 0.34).

**Figure 5:**
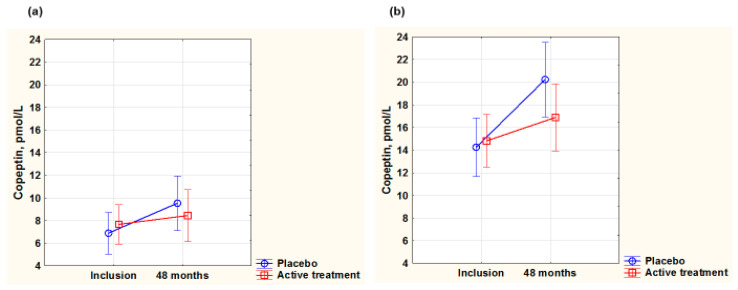
Concentration of copeptin evaluated in the active treatment group versus placebo group at inclusion and after 48 months in the female group (**a**) and in the male group (**b**). Note: (**a**) Current effect: F(1,116) = 2.60; *p* = 0.11. Active treatment: *n* = 62; placebo: *n* = 56. (**b**) Current effect: F(1,96) = 4.89; *p* = 0.03. Active treatment: *n* = 55; placebo: *n* = 45. Evaluation performed by use of repeated measures of variance methodology. Vertical bars denote 0.95 confidence interval. Blue curve: placebo; red curve: active treatment.

To validate the obtained results, a repeated measure of variance analysis (including participants surviving during the 48 months) was performed. After 48 months, in females, there was no significant difference in copeptin concentrations between the two groups (F(1,116) = 2.60; *p* = 0.11) (Figure 5a,b), while in the male group, a significant difference between the active treatment and the placebo group was noted, where the placebo group had a more increased value (F(1,96)= 4.89; *p* = 0.03).

#### 3.6.2. N-Terminal proBNP in Males and Females

As NT-proBNP is a biomarker for cardiac wall tension, this biomarker was evaluated in the two sexes in our study population. At inclusion, males had higher NT-proBNP values than females (Table 3).

Neither in females nor in males were there any differences between the active treatment groups and the placebo groups at baseline (females: active: 353 ng/L; placebo: 491 ng/L; *p* = 0.12; males: active: 727 ng/L; placebo 544 ng/L; *p* = 0.41). After 48 months, no significant differences were observed between the active and placebo groups in the female group (females: active: 356 ng/L; placebo 463 ng/L; *p* = 0.21). However, in the male group, a significantly higher NT-proBNP concentration was found in the placebo group, compared to the active treatment group (males: active: 531 ng/L; placebo: 855 ng/L; *p* = 0.04).

**Figure 6:**
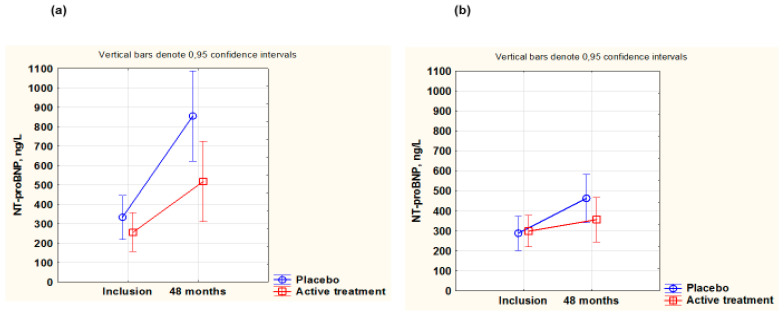
Concentration of NT-proBNP evaluated in the active treatment group versus placebo group at inclusion and after 48 months in the male group (**a**) and in the female group (**b**). Note: (**a**) Current effect: F(1,98) = 4.20; *p* = 0.04. Active treatment: *n* = 57; placebo: *n* = 44. (**b**) Current effect: F(1,113) = 5.32; *p* = 0.02. Active treatment: *n* = 62; placebo: *n* = 53. Evaluation performed by use of repeated measures of variance methodology. Vertical bars denote 0.95 confidence interval. Blue curve: placebo; red curve: active treatment.

Upon applying repeated measures of variance to the female group, a higher value at 48 months in the placebo group could be noted compared with the active treatment group (F(1,113)= 5.32; *p* = 0.02) (Figure 6a,b). Equally, in the male group at 48 months, a higher value in the placebo group compared with the active treatment group was noted (F(1,98)= 4.20; *p* = 0.04). At 48 months, the NT-proBNP values of the females appeared to be generally lower than the values observed in the male group, probably due to a higher prevalence of heart failure in the male group. The lower NT-proBNP values at inclusion seen for males and females in Figure 6a,b compared to the values in the total population at inclusion (Table 3) are due to a higher mortality during the intervention period among participants with the higher NT-proBNP values.

#### 3.6.3. Renal Function in MALES and Females

Evaluation of the renal function was performed in the two groups using the Chronic Kidney Disease Epidemiology Collaboration (CKD-Epi) algorithm, in which both creatinine and cystatin-C are included.

At inclusion, there were no differences in the glomerular filtration rate (GFR) between females and males (females: 61.5 mL/min/1.73 m^2^; males: 61.7 mL/min/1.73 m^2^; *p*= 0.94).

In the female group, no difference in GFR was observed at inclusion (active: 59.7 mL/min/1.73 m^2^; placebo: 63.3 mL/min/1.73 m^2^; *p* = 0.32). However, after 48 months, a highly significant difference between the active and the placebo groups was observed (active: 76.1 mL/min/1.73 m^2^; placebo: 62.0 mL/min/1.73 m^2^; *p* = 0.0002), indicating an improved filtration rate in the active group. In the male group, no difference in GFR was noted at inclusion (active: 63 mL/min/1.73 m^2^; placebo: 59.9 mL/min/1.73 m^2^; *p* = 0.32). After 48 months, also in the actively treated males, a significantly higher GFR was seen, indicating improvement, compared with the placebo group (active: 74.7 mL/min/1.73 m^2^; placebo: 65.6 mL/min/1.73 m^2^; *p* = 0.02).

**Figure 7:**
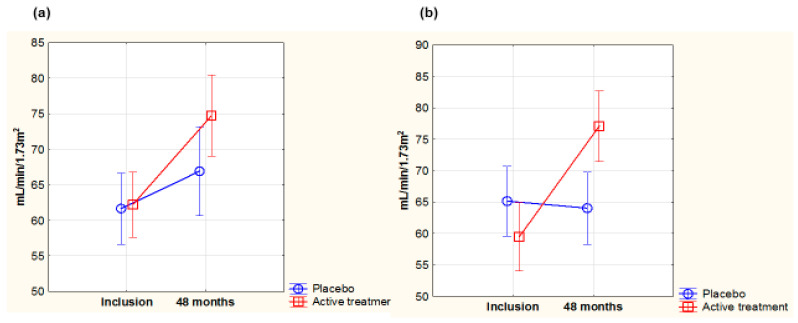
Concentration of glomerular filtration rate according to the CKD-Epi algorithm evaluated in the active treatment group versus placebo group at inclusion and after 48 months in the female group (**a**) and in the male group (**b**). Note: (**a**) Current effect: F(1,86) = 23.4; *p* = 0.00001. Active treatment: *n* = 53; placebo: *n* = 52. (**b**) Current effect: F(1,81) = 3.75; *p* = 0.056. Active treatment: *n* = 57; placebo: *n* = 43. Evaluation performed by use of repeated measures of variance methodology. Vertical bars denote 0.95 confidence interval. Blue curve: placebo; red curve: active treatment.

To validate these results, repeated measures of variance analyses were performed on female and male participants surviving the intervention period (Figure 7a,b).

The observed increase in GFR from baseline in the supplemented groups was almost the same and significant for both females (*p* < 0.0001) and males (*p* = 0.0005). In the female group there was a highly significant difference with higher GFR in the active treatment group compared with the placebo group (*p* < 0.0001), whereas in the male group, a trend for an increased GFR in the active treatment group compared with the placebo group was seen (*p* = 0.056).

An ANCOVA analysis of the covariates in the female group using CKD-Epi after 48 months as a dependent variable revealed that besides CKD-Epi at inclusion and active treatment, hypertension and CRP also influenced the dependent variable (Table 4).

Performing the same analysis in the male group showed that besides CKD-Epi at inclusion, and NYHA functional class III, active treatment, when adjusted for several well-known covariates influencing renal function, also had a significant influence on the dependent variable (Table 5). Notably, while smoking prevalence in males was three times higher than that of females, smoking did not significantly impact eGFR at 48 months. The beneficial effects of supplementation on the female and the male groups were similar and of almost the same magnitude.

## 4. Discussion

The main findings in this study on elderly people deficient in selenium were that, regarding CV health, women to a greater extent than men seemed to benefit from supplementation with selenium and CoQ_10_. Furthermore, the intervention had a protective effect on CV mortality both in female and male participants with and without diagnosed IHD at inclusion. The impact was equal in females and males with IHD but seemed greater in females without IHD. While active treatment appeared to increase survival during the whole follow-up period in those with IHD, increased survival in those without IHD kicked in past the active intervention period, from about the fourth year and onwards of the follow-up.

We have reported in previous publications [5,32,34] that this elderly population has benefited from supplementation with selenium and Q_10_ concerning CV health and health aspects. However, potential sex-specific differences in the effects of supplementations with the two substances have not been analysed or discussed. Therefore, in the present study, we applied sex-based stratification to analyse the effects in the sub-analyses from the previously reported randomized clinical trial (RCT) [1]. As mortality is undoubtedly a hard endpoint, we evaluated CV mortality in the two sexes separately. As the result showed significant differences in mortality between females and males, but also between active treatment versus placebo groups, a separate baseline evaluation of the two sexes was indicated. The results highlight important differences between males and females that may impact CV mortality.

Already at inclusion, the male group exhibited a higher rate of smoking and more signs of IHD, with the resulting impaired cardiac function and increased cardiac wall tension. This accords with results reported in several studies in the literature regarding elderly patients with coronary artery disease [35]. That smoking is more common among males than in females is a frequent observation; however, it is also reported that females who smoke are exposed to a higher cardiovascular risk, especially at younger ages [36,37]. In general, it has been shown that females present with coronary artery disease about 10 years later than males [38,39]. One of the important differences in atherosclerotic plaque structure is that females usually have a more fibrotic plaque, whereas males have a more atheromatous plaque [40]. The status at baseline of the two sexes therefore concurs with the literature. From the baseline characteristics, both HDL and LDL presented with higher levels in the female group. This is also in accordance with the literature, where females regularly present with higher lipid levels and where oestrogens are believed to have a protective effect [37] that disappears after menopause [41].

It is also important to follow the development of two of the major risk factors in the development of atherosclerosis: inflammation and oxidative stress. We found differences between the two sexes both in inflammation (CRP, P-selectin, and osteopontin), and in oxidative stress (copeptin). The male group appeared to have developed increased inflammation and oxidative stress, as compared with the females, both at inclusion and during the follow-up period. In the literature, reports indicate that males have a higher concentration of copeptin than females [42]. It has been proposed that copeptin has anti-oxidative properties and is linked to oestrogen production, which decreases after menopause [43]. However, as regards inflammation, reports indicate that in a normal elderly population, females present with higher concentrations of inflammation biomarkers than males [44]. In the present study population, however, we found signs of the opposite, that the male group presented with a higher degree of inflammation than the females, which might indicate increased underlying atherosclerotic activity among the males, at least in our study groups.

Another interesting aspect of the two groups was the obtained levels of NT-proBNP, a peptide that is synthesised in the cardiomyocytes in response to increased wall tension. In the literature, it is reported that in a normal population, the reference values increase with age, and that females have higher concentrations than males. However, the reference values are considerably lower than those seen in our study population, especially in the male group [45]. It is also interesting to note that in the population of both sexes surviving for four years, the level of NT-proBNP increased especially in the placebo group, and especially in the males, as an indicator of increased wall tension, probably because of a previous ischaemic event. As the NT-proBNP elimination rate is dependent on renal function, it is also important to consider renal function in the two groups. From these results on renal function it is obvious that in both sexes on active treatment with selenium and CoQ_10_, renal function improved hence, the increase seen in NT-proBNP could not be due to impaired renal function. In fact, the renal function at inclusion and as a response to the intervention seemed to be similar in females and males.

It is tempting to suggest that the stronger positive impact in women than in men of the supplementation with selenium and CoQ_10_ in our study is because at inclusion the males already had a more advanced picture of CV risk factors and disease than the females. The difference in IHD prevalence exemplifies this important variance. During the follow-up period, this influenced the different progression of the above-described markers. Based on the considerations above, we hypothesised that supplementation would have less effect on CV mortality in participants with more advanced CVD, such as those with IHD at inclusion. However, even among those with IHD we observed significant effects of the supplementation in the female group and an almost significant effect (*p* = 0.06) in the male group, probably due to the limited sample size. After combining the two sexes, the Kaplan–Meier analysis revealed a significant effect of the supplementation on CV mortality in those with IHD at inclusion. It is suggested that the supplementation with selenium and CoQ_10_ also elicited positive effects in those already diseased, but the impact may have been less prominent. The early positive effect on those with IHD might indicate that supplementation reduced oxidative stress and inflammation, causing an anti-atherogenic action and impaired disease progression.

Another interesting aspect of the IHD group is the fact that based on the criteria to be classified as belonging to the IHD group, only those with symptoms or pathological ECGs were identified, which means that those with early-stage IHD but still without symptoms would be classified as not having IHD, even though some participants in this asymptomatic group were probably present among those who died from CV-related causes during the 10-year follow-up. It is also highly probable that there was male dominance in this group.

Evaluating the impact of supplementation on CV mortality in the two sexes that did not have IHD at inclusion showed that there was a significantly lower CV mortality in both sexes in the groups on active treatment. This was apparently lower among females and, as seen in the Kaplan–Meier analysis, kicked in at a later stage. Hence, it seems likely that the effect of the supplementation would be more beneficial when given before or at an earlier stage of CVD development. For those without IHD, the greater beneficial effect seen in females compared with males was likely due to more advanced CV changes in addition to a higher prevalence of smokers in males (14.1 vs. 4.4%). Smokers were included in the study groups, and a considerable sex difference in smoking prevalence was noted, in agreement with the lifestyle habits of the population during the time of the study initiation. This difference was accounted for in the analyses.

In an earlier sub-study of this population, we found that the supplementation prevented leukocyte telomere attrition in both sexes, though with a seemingly stronger impact on telomere length preservation in women. Less telomere shortening was also associated with longer survival from CV mortality [46].

## 5. Novelty with This Sub-Analysis

This sub-analysis presents for the first time an analysis of the impact of sex on the benefit of selenium and CoQ_10_ supplementation on CVD and CV mortality. Substantial differences in mortality could be demonstrated between males and females, but after a more in-depth analysis, it could be shown that the male group was, at inclusion, already significantly more diseased with, for example, IHD, compared with the females. This could be seen regarding inflammation and oxidative stress, but also in the amount of impaired cardiac systolic function. The supplementation with selenium and CoQ_10_ did also have an effect in the already diseased group; however, it was more effective in those without disease.

From a disease prevention perspective, it is therefore important to correct deficiencies in selenium and CoQ_10_ before or at an early stage of CVD development.

We think the points above would be of clinical interest for those handling patients (especially those at risk for CVD) in their daily practice and constitute real novelties for the colleagues working in this field of medicine.

## 6. Limitations

The limited sample population utilised in this study may contribute to the uncertainty surrounding the results obtained. Evaluations were conducted using a two-step procedure, which was employed in several analyses to bolster the internal validity and reliability of the findings. Given the constrained sample size and the exploratory nature of the study, the results should be regarded as generating hypotheses rather than providing definitive conclusions.

Additionally, a significant limitation of the study is the inclusion of only one treatment group that received a combination of selenium and CoQ_10_, alongside a placebo group. This restriction was imposed due to resource limitations during the original study design period from 2002 to 2003. Furthermore, the study population is characterised by a narrow age range of elderly Caucasians residing in Scandinavia and a selenium-deficient population. Consequently, the findings of this research may not be generalisable to other age groups or populations with differing selenium status and varying characteristics.

## 7. Conclusions

In a sub-analysis from an RCT where supplementation with selenium and CoQ10 were given for four years, the females showed both a stronger effect of the supplementation and a reduced rate of cardiovascular mortality compared with the males. These sex-specific differences may be related to more advanced CVD in the males at inclusion, such as diagnosed IHD and signs of heart failure, as well as higher inflammation and oxidative stress. Importantly, in both the male and female participants with IHD, supplementation improved CV survival. In those without IHD at inclusion, the supplementation appeared more efficient, but the effect was first seen after four years of follow-up and was stronger in females. Although the supplementation seemed to result in a general benefit, it appears that CV mortality is more effectively reduced when supplementation occurs at an earlier stage of CVD development as a measure of disease prevention. To this end, early diagnostics of CV function and the correction of a diagnosed selenium and CoQ10 deficiency may represent the most efficient and cost-effective health-supporting measure and meaningful population-wide strategy.

## Figures and Tables

**Table 1:** Baseline characteristics of the study population receiving active treatment or placebo during an intervention time of four years.

	Active	Placebo	*p*-Value
*n*	221	222	
Age years mean (SD)	77.0 (3.6)	77.3 (3.4)	
Males/females *n*	115/106	110/112	
**History**			
Smokers (present), *n* (%)	21 (9.5)	20 (9.0)	0.86
Diabetes, *n* (%)	47 (21.3)	48 (21.6)	0.93
Hypertension, *n* (%)	158 (71.5)	168 (75.7)	0.32
NYHA class III, *n* (%)	41 (18.6)	47 (21.2)	0.49
IHD, *n* (%)	47 (21.3)	53 (23.9)	0.51
**Medications**			
ACEI, *n* (%)	37 (16.7)	54 (24.3)	0.03
ARB, *n* (%)	10 (4.5)	13 (5.9)	0.53
Betablockers, *n* (%)	80 (36.2)	69 (31.1)	0.25
Anticoagulants, *n* (%)	26 (11.8)	34 (15.3)	0.27
Diuretics, *n* (%)	70 (31.7)	88 (39.6)	0.08
Statins, *n* (%)	45 (20.4)	51 (23.0)	0.50
**Examinations**			
EF < 40%, *n* (%)	16 (7.2)	17 (7.7)	0.87
s-Se µg/L, pre-intervention, mean, (SD)	67.7 (14.8)	67.6 (15.8)	0.98
s-Se µg/L, post-intervention, mean, (SD)	210.2 (60.7)	71.9 (25.6)	<0.00001

Note: ACEI: ACE inhibitors; ARB: angiotensin receptor blockers; EF: ejection fraction; IHD: ischaemic heart disease; NYHA: New York Heart Association functional class; SD: standard deviation; s-Se: serum selenium. Values are means ± SDs or frequency (percent). Student’s unpaired two-sided *t*-test was used for continuous variables, and the chi-square test was used for analysis of one discrete variable.

**Table 2:** Cox proportional hazard regression analysis evaluating risk of CV mortality and supplementation of selenium and coenzyme Q_10_ combined in a multivariate model after 10 years of follow-up and after 4 years of intervention with (**a**) a female population and (**b**) a male population.

**(a)**					
**Variables**	**Beta Value**	**Hazard Ratio**	**T-Value**	**95% CI**	***p*-Value**
**Hypertension**	0.48	1.62	1.28	0.77–3.41	0.20
**Diabetes**	0.72	2.05	2.31	1.12–3.77	0.02
**Ischaemic heart disease, incl.**	1.18	3.26	3.70	1.74–6.11	<0.0001
**Smoker**	0.60	1.82	0.98	0.55–6.08	0.33
**NT-proBNP Q5 females, incl.**	1.12	3.06	3.47	1.63–5.76	<0.0003
**Active treatment**	−1.22	0.29	−3.60	0.15–0.57	<0.0005
**Obstr pulm disease**	−0.03	0.97	−0.08	0.46–2.07	0.94
**(b)**					
**Variables**	**Beta Value**	**Hazard Ratio**	**T-Value**	**95% CI**	***p*-Value**
**Hypertension**	0.17	1.18	0.63	0.70–1.99	0.53
**Diabetes**	0.48	1.61	1.95	1.00–2.60	0.05
**Ischaemic heart disease, incl.**	0.40	1.49	1.69	0.94–2.35	0.09
**Smoker**	0.90	2.47	3.36	1.46–4.18	0.0008
**NT-proBNP Q5 males, incl.**	1.23	3.43	4.96	2.11–5.57	<0.0001
**Active treatment**	−0.49	0.61	−2.14	0.39–0.96	0.03
**Obstr pulm disease**	0.30	1.35	1.00	0.75–2.43	0.32

Note: NT-proBNP: N-terminal fragment of B-type natriuretic peptide; Q: quintile.

**Table 4:** Analysis of covariance in the female group using CKD-Epi as dependent variable after 48 months.

Effects	Degrees of Freedom	F	*p*	Mean Square
Intercept	1	3.14	0.08	760
Age	1	0.79	0.38	190
Smoking	1	0.06	0.81	13
Hypertension	1	5.86	0.017	1420
Diabetes	1	0.99	0.32	239
IHD	1	0.06	0.81	14
NYHA III	1	0.08	0.78	19
NT-proBNP Q4 incl	1	0.13	0.72	32
s-selenium µg/L, incl	1	0.32	0.58	77
CRP, mg/L, incl	1	6.5	0.01	1579
CKD-Epi incl mL/min/1.73 m^2^	1	30.29	<0.0001	7333
Active treatment	1	21.84	<0.0001	5287
Error	92			242

Note: IHD: ischaemic heart disease; NYHA: New York Heart Association functional class III; Q: quartile. Mult. R: 0.68; Adj R^2^: 0.40.

**Table 5:** Analysis of covariance in the male group using CKD-Epi as dependent variable after 48 months.

Effects	Degrees of Freedom	F	*p*	Mean Square
Intercept	1	2.59	0.11	604
Age	1	1.07	0.30	249
Smoking	1	0.59	0.45	137
Hypertension	1	2.99	0.09	696
Diabetes	1	0.91	0.34	211
IHD	1	1.36	0.25	317
NYHA III	1	4.49	0.04	1047
NT-proBNP Q4 incl	1	0.02	0.88	5.76
s-selenium µg/L, incl	1	0.01	0.90	3
CRP, mg/L, incl	1	0.04	0.84	10
CKD-Epi incl ml/mim/1.73 m^2^	1	34.8	<0.0001	8115
Active treatment	1	4.22	0.04	983
Error	85			233

Note: IHD: ischaemic heart disease; NYHA: New York Heart Association functional class III; Q: quartile. Mult. R: 0.66; Adj R^2^: 0.36.

## Data Availability

Under Swedish Law, the authors cannot share the data used in this study and cannot conduct any further research other than what is specified in the ethical permissions application. For inquiries about the data, researchers should first contact the owner of the database, the University of Linköping. Please contact the corresponding author with requests for and assistance with data. If the university approves the request, researchers can submit an application to the Regional Ethical Review Board for the specific research question that the researcher wants to examine.

## Supplementary Materials

The following supporting information can be downloaded at https://www.mdpi.com/article/10.3390/antiox14060685/s1.

## Abbreviations

ACE: angiotensin converting enzyme; ANCOVA: analysis of covariance; ARB: angiotension receptor blockers; B.I.D: bis in die = two times a day; BMI: body mass index; CoQ10: coenzyme Q_10_; CKD-EPI: Chronic Kidney Disease Epidemiology Collaboration; CVD: cardiovascular disease; ECG: electrocardiogram: EF: ejection fraction; ELISA: enzyme-linked immunosorbent assay; GFR: glomerular filtration rate; IHD: ischaemic heart disease; NT-proBNP: N-terminal fragment of B-type natriuretic peptide; NYHA: New York Heart Association functional class; RCT: randomized clinical trial; SD: standard deviation.

## References

[1] Alehagen U., Johansson P., Bjornstedt M., Rosen A., Dahlstrom U. (2013). Cardiovascular mortality and N-terminal-proBNP reduced after combined selenium and coenzyme Q10 supplementation: A 5-year prospective randomized double-blind placebo-controlled trial among elderly Swedish citizens. Int. J. Cardiol..

[2] Alehagen U., Aaseth J., Johansson P. (2015). Reduced Cardiovascular Mortality 10 Years after Supplementation with Selenium and Coenzyme Q10 for Four Years: Follow-Up Results of a Prospective Randomized Double-Blind Placebo-Controlled Trial in Elderly Citizens. PLoS ONE.

[3] Alehagen U., Aaseth J., Alexander J., Johansson P. (2018). Still reduced cardiovascular mortality 12 years after supplementation with selenium and coenzyme Q10 for four years: A validation of previous 10-year follow-up results of a prospective randomized double-blind placebo-controlled trial in elderly. PLoS ONE.

[4] Alehagen U., Lindahl T.L., Aaseth J., Svensson E., Johansson P. (2015). Levels of sP-selectin and hs-CRP Decrease with Dietary Intervention with Selenium and Coenzyme Q10 Combined: A Secondary Analysis of a Randomized Clinical Trial. PLoS ONE.

[5] Alehagen U., Alexander J., Aaseth J., Larsson A. (2019). Decrease in inflammatory biomarker concentration by intervention with selenium and coenzyme Q10: A subanalysis of osteopontin, osteoprotergerin, TNFr1, TNFr2 and TWEAK. J. Inflamm..

[6] Regitz-Zagrosek V., Kararigas G. (2017). Mechanistic Pathways of Sex Differences in Cardiovascular Disease. Physiol. Rev..

[7] Kane A.E., Howlett S.E. (2018). Differences in Cardiovascular Aging in Men and Women. Adv. Exp. Med. Biol..

[8] Merz A.A., Cheng S. (2016). Sex differences in cardiovascular ageing. Heart.

[9] Peters S.A., Huxley R.R., Woodward M. (2013). Comparison of the sex-specific associations between systolic blood pressure and the risk of cardiovascular disease: A systematic review and meta-analysis of 124 cohort studies, including 1.2 million individuals. Stroke.

[10] Safiri S., Karamzad N., Singh K., Carson-Chahhoud K., Adams C., Nejadghaderi S.A., Almasi-Hashiani A., Sullman M.J.M., Mansournia M.A., Bragazzi N.L. (2022). Burden of ischemic heart disease and its attributable risk factors in 204 countries and territories, 1990–2019. Eur. J. Prev. Cardiol..

[11] Yeo W.J., Abraham R., Surapaneni A.L., Schlosser P., Ballew S.H., Ozkan B., Flaherty C.M., Yu B., Bonventre J.V., Parikh C.R. (2024). Sex Differences in Hypertension and Its Management Throughout Life. Hypertension.

[12] Rayman M.P. (2012). Selenium and human health. Lancet.

[13] Fairweather-Tait S.J., Bao Y., Broadley M.R., Collings R., Ford D., Hesketh J.E., Hurst R. (2011). Selenium in human health and disease. Antioxid. Redox. Signal.

[14] Kafai M.R., Ganji V. (2003). Sex, age, geographical location, smoking, and alcohol consumption influence serum selenium concentrations in the USA: Third National Health and Nutrition Examination Survey, 1988–1994. J. Trace Elem. Med. Biol..

[15] Bleys J., Navas-Acien A., Laclaustra M., Pastor-Barriuso R., Menke A., Ordovas J., Stranges S., Guallar E. (2009). Serum selenium and peripheral arterial disease: Results from the national health and nutrition examination survey, 2003–2004. Am. J. Epidemiol..

[16] Van Cauwenbergh R., Robberecht H., Van Vlaslaer V., Deelstra H. (2004). Comparison of the serum selenium content of healthy adults living in the Antwerp region (Belgium) with recent literature data. J. Trace Elem. Med. Biol..

[17] Burri J., Haldimann M., Dudler V. (2008). Selenium status of the Swiss population: Assessment and change over a decade. J. Trace Elem. Med. Biol..

[18] Letsiou S., Nomikos T., Panagiotakos D., Pergantis S.A., Fragopoulou E., Antonopoulou S., Pitsavos C., Stefanadis C. (2009). Serum total selenium status in Greek adults and its relation to age. The ATTICA study cohort. Biol. Trace Elem. Res..

[19] Spina A., Guallar E., Rayman M.P., Tigbe W., Kandala N.B., Stranges S. (2013). Anthropometric indices and selenium status in British adults: The U.K. National Diet and Nutrition Survey. Free Radic. Biol. Med..

[20] Galan-Chilet I., Tellez-Plaza M., Guallar E., De Marco G., Lopez-Izquierdo R., Gonzalez-Manzano I., Carmen Tormos M., Martin-Nunez G.M., Rojo-Martinez G., Saez G.T. (2014). Plasma selenium levels and oxidative stress biomarkers: A gene-environment interaction population-based study. Free Radic. Biol. Med..

[21] Bullon P., Roman-Malo L., Marin-Aguilar F., Alvarez-Suarez J.M., Giampieri F., Battino M., Cordero M.D. (2015). Lipophilic antioxidants prevent lipopolysaccharide-induced mitochondrial dysfunction through mitochondrial biogenesis improvement. Pharmacol. Res..

[22] Lee B.J., Tseng Y.F., Yen C.H., Lin P.T. (2013). Effects of coenzyme Q10 supplementation (300 mg/day) on antioxidation and anti-inflammation in coronary artery disease patients during statins therapy: A randomized, placebo-controlled trial. Nutr. J..

[23] Kalen A., Appelkvist E.L., Dallner G. (1989). Age-related changes in the lipid compositions of rat and human tissues. Lipids.

[24] Xia L., Nordman T., Olsson J.M., Damdimopoulos A., Bjorkhem-Bergman L., Nalvarte I., Eriksson L.C., Arner E.S., Spyrou G., Bjornstedt M. (2003). The mammalian cytosolic selenoenzyme thioredoxin reductase reduces ubiquinone. A novel mechanism for defense against oxidative stress. J. Biol. Chem..

[25] Lam C.S.P., Arnott C., Beale A.L., Chandramouli C., Hilfiker-Kleiner D., Kaye D.M., Ky B., Santema B.T., Sliwa K., Voors A.A. (2019). Sex differences in heart failure. Eur. Heart J..

[26] Alehagen U., Johansson P., Bjornstedt M., Rosen A., Post C., Aaseth J. (2016). Relatively high mortality risk in elderly Swedish subjects with low selenium status. Eur. J. Clin. Nutr..

[27] Morgenthaler N.G., Struck J., Alonso C., Bergmann A. (2006). Assay for the measurement of copeptin, a stable peptide derived from the precursor of vasopressin. Clin. Chem..

[28] McFadden E.C., Hirst J.A., Verbakel J.Y., McLellan J.H., Hobbs F.D.R., Stevens R.J., O’Callaghan C.A., Lasserson D.S. (2018). Systematic Review and Metaanalysis Comparing the Bias and Accuracy of the Modification of Diet in Renal Disease and Chronic Kidney Disease Epidemiology Collaboration Equations in Community-Based Populations. Clin. Chem..

[29] Alexander J., Olsen A.K. (2023). Selenium—A scoping review for Nordic Nutrition Rcommendations 2023. Food Nutr. Res..

[30] Letsiou S., Damigou E., Nomikos T., Pergantis S.A., Pitsavos C., Panagiotakos D., Antonopoulou S. (2024). Deciphering the associations of selenium distribution in serum GPx-3 and selenoprotein P with cardiovascular risk factors in a healthy population with moderate levels of selenium: The ATTICA study. J. Trace Elem. Med. Biol..

[31] Perri G., Mathers J.C., Martin-Ruiz C., Parker C., Walsh J.S., Eastell R., Demircan K., Chillon T.S., Schomburg L., Robinson L. (2024). Selenium status and its determinants in very old adults: Insights from the Newcastle 85+ Study. Br. J. Nutr..

[32] Alehagen U., Alexander J., Aaseth J. (2016). Supplementation with Selenium and Coenzyme Q10 Reduces Cardiovascular Mortality in Elderly with Low Selenium Status. A Secondary Analysis of a Randomised Clinical Trial. PLoS ONE.

[33] Alexander J., Aaseth J.O., Schomburg L., Chillon T.S., Larsson A., Alehagen U. (2024). Circulating Glutathione Peroxidase-3 in Elderly-Association with Renal Function, Cardiovascular Mortality, and Impact of Selenium and Coenzyme Q(10) Supplementation. Antioxidants.

[34] Alehagen U., Aaseth J., Alexander J., Svensson E., Johansson P., Larsson A. (2018). Less fibrosis in elderly subjects supplemented with selenium and coenzyme Q10-A mechanism behind reduced cardiovascular mortality?. Biofactors.

[35] Nunez J., Lorenzo M., Minana G., Palau P., Monmeneu J.V., Lopez-Lereu M.P., Gavara J., Marcos-Garces V., Rios-Navarro C., Perez N. (2021). Sex differences on new-onset heart failure in patients with known or suspected coronary artery disease. Eur. J. Prev. Cardiol..

[36] Vasiljevic Z., Scarpone M., Bergami M., Yoon J., van der Schaar M., Krljanac G., Asanin M., Davidovic G., Simovic S., Manfrini O. (2021). Smoking and sex differences in first manifestation of cardiovascular disease. Atherosclerosis.

[37] Yoon Y.H., Park G.M., Lee J.Y., Lee J.H., Roh J.H., Kim T.O., Lee P.H., Choe J., Kim Y.H., Lee S.W. (2023). Relationship between sexual differences and cardiovascular risk factors in the prevalence of asymptomatic coronary disease. Int. J. Cardiol..

[38] Maas A.H., Appelman Y.E. (2010). Gender differences in coronary heart disease. Neth. Heart J..

[39] Canto J.G., Rogers W.J., Goldberg R.J., Peterson E.D., Wenger N.K., Vaccarino V., Kiefe C.I., Frederick P.D., Sopko G., Zheng Z.J. (2012). Association of age and sex with myocardial infarction symptom presentation and in-hospital mortality. JAMA.

[40] Sakkers T.R., Mokry M., Civelek M., Erdmann J., Pasterkamp G., Diez Benavente E., den Ruijter H.M. (2023). Sex differences in the genetic and molecular mechanisms of coronary artery disease. Atherosclerosis.

[41] Shah T., Palaskas N., Ahmed A. (2016). An Update on Gender Disparities in Coronary Heart Disease Care. Curr. Atheroscler. Rep..

[42] Schill F., Persson M., Engstrom G., Melander O., Enhorning S. (2021). Copeptin as a marker of atherosclerosis and arteriosclerosis. Atheroscler..

[43] Mendoza-Nunez V.M., Beristain-Perez A., Perez-Vera S.P., Altamirano-Lozano M.A. (2010). Age-related sex differences in glutathione peroxidase and oxidative DNA damage in a healthy Mexican population. J. Womens Health.

[44] Lodge S., Masuda R., Nitschke P., Beilby J.P., Hui J., Hunter M., Yeap B.B., Millet O., Wist J., Nicholson J.K. (2025). NMR spectroscopy derived plasma biomarkers of inflammation in human populations: Influences of age, sex and adiposity. PLoS ONE.

[45] Macheret F., Boerrigter G., McKie P., Costello-Boerrigter L., Lahr B., Heublein D., Sandberg S., Ikeda Y., Cataliotti A., Bailey K. (2011). Pro-B-type natriuretic peptide(1-108) circulates in the general community: Plasma determinants and detection of left ventricular dysfunction. J. Am. Coll. Cardiol..

[46] Opstad T.B., Alexander J., Aaseth J.O., Larsson A., Seljeflot I., Alehagen U. (2022). Selenium and Coenzyme Q(10) Intervention Prevents Telomere Attrition, with Association to Reduced Cardiovascular Mortality-Sub-Study of a Randomized Clinical Trial. Nutrients.

