# Supplementary figures and images for: Selenium and Coenzyme Q10 Supplementation and Sex Differences in Cardiovascular Mortality Results from a Prospective Randomized Double-Blind Placebo-Controlled Trial in Elderly People Low in Selenium
Source: Antioxidants (Basel). 2025 Jun 5;14(6):685. doi: 10.3390/antiox14060685 (PMC12190002; doi:10.3390/antiox14060685)

## Supplemental files

Supplemental Figure S1

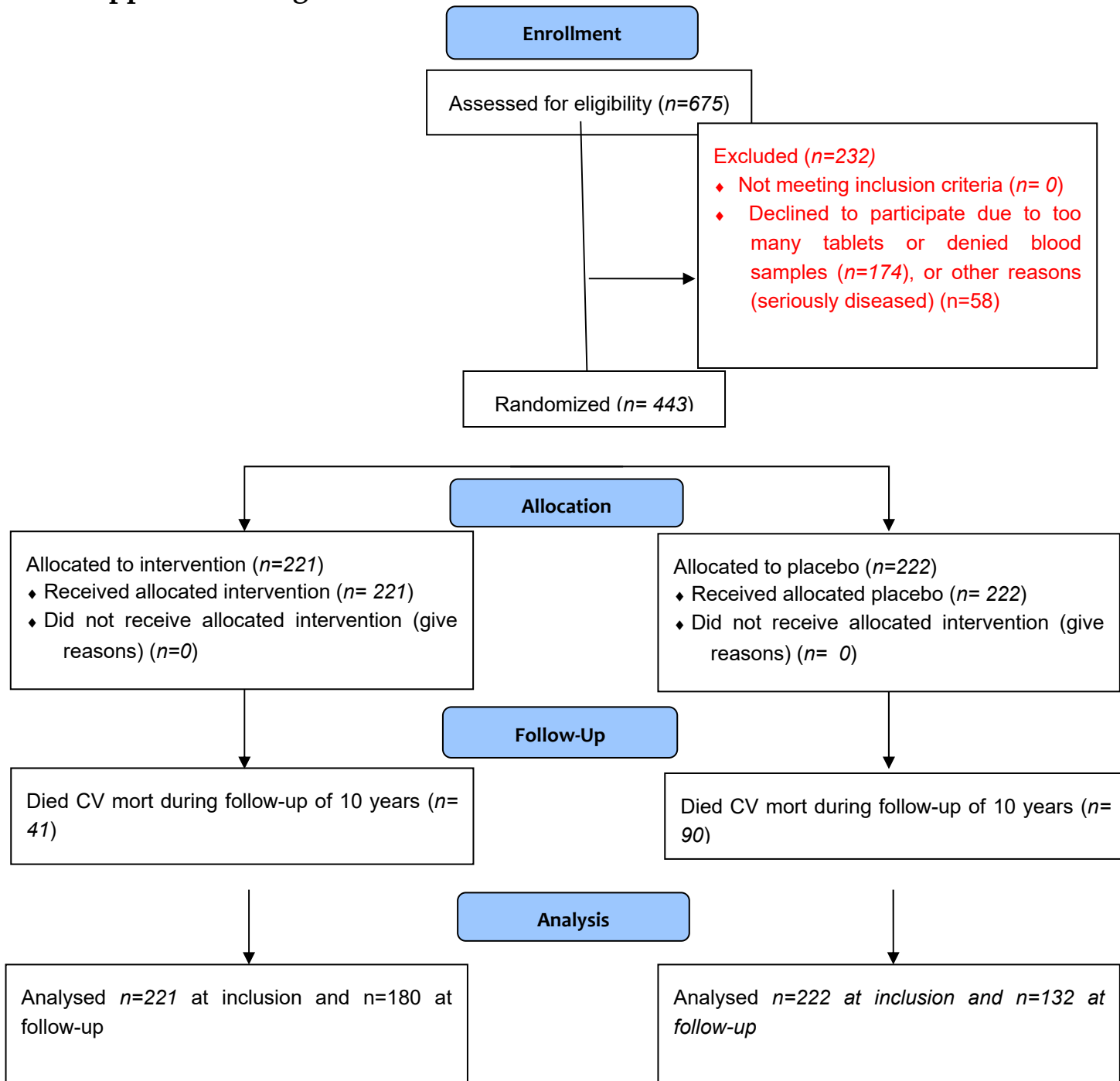

Supplement: Supplementary file 1 [file antioxidants-14-00685-s001.zip › antioxidants-3627075-SM.pdf]
